# Study of karyotype and constitutive heterochromatin of *Mansonia* spp. (Diptera: Culicidae) in the area surrounding the Jirau Hydroelectric Dam, Rondônia, Brazil

**DOI:** 10.1590/0037-8682-0095-2022

**Published:** 2023-03-06

**Authors:** Míriam Silva Rafael, Sabrina da Fonseca Meireles, Vânia da Costa Ferreira, Wanderli Pedro Tadei, Rosemary Aparecida Roque

**Affiliations:** 1 Instituto Nacional de Pesquisas da Amazônia, Pós-Graduação em Genética, Conservação e Biologia Evolutiva, Manaus, AM, Brasil.; 2 Usina Hidrelétrica Jirau, Energia Sustentável do Brasil, Porto Velho, RO, Brasil.; 3 Instituto Nacional de Pesquisas da Amazônia, Coordenação de Sociedade, Ambiente e Saúde, Laboratório de Malária e Dengue, Manaus, AM, Brasil.

**Keywords:** Mosquito brain ganglia, Chromosomal structure, Filariasis

## Abstract

**Background:**

*Mansonia* mosquitoes transmit arboviruses to humans. This study describes the karyotypes and C-banding of *Mansonia humeralis*, *Mansonia titillans, Mansonia pseudotitillans*, and *Mansonia indubitans.*

**Methods:**

From the 202 larvae, the brain ganglia were dissected (n=120) for the preparation of slides. Twenty slides with well-distended chromosomes for each species (10 for karyotyping and 10 for C-banding) were selected for further study.

**Results:**

The haploid genome and the average lengths of the chromosomal arms differed in relation to the centromere between species, and intraspecific differences also occurred in the distribution of the C-bands.

**Conclusions:**

These results are useful for better understanding of the chromosomal variability of *Mansonia* mosquitoes.

Mosquitoes of the genus *Mansonia* (Diptera: Culicidae) comprise 25 species which are taxonomically identified in two subgenera, *Mansonia Blanchard*, 1901 and *Mansonioides* Theobald, both of which are widely distributed in the African continent and the Neotropical region^1^. These mosquitoes have similar external morphological characteristics, which makes it difficult to correctly identify them in a taxonomic sense. In the Amazon, the species *Mansonia titillans* (Walker, 1848), *Mansonia pseudotitillans* (Theobald, 1901)*, Mansonia amazonensis* (Theobald, 1901), *Mansonia flaveola* (*Coquillett,* 1906), *Mansonia humeralis* (Dyar & Knab*,* 1916), *Mansonia indubitans* (Dyar & Shannon, 1925), and *Mansonia dyari*Belkin, Heinemann & Page, 1970 have been reported^1^.

*Mansonia* mosquitoes are hematophagous arthropods and vectors of the nematode helminths *Wuchereria bancrofti*, *Brugia malayi,* and *Brugia timori*, which are known as human lymphatic filariases^1^ ^,^ ^2^. *Mansonia titillans* is a primary vector of equine encephalitis virus in Venezuela and Guatemala^2^, and has been found naturally infected with West Nile virus in the US states of Florida and Louisiana^3^. *Mansonia titillans* and *Ma. humeralis* were captured while performing hematophagy on humans and cattle, respectively^2^ ^,^ ^3^. *Mansonia indubitans* females are daytime biters with aggressive and voracious behavior^4^ ^,^ ^6^. *Mansonia humeralis* has been found naturally infected with dengue and Mayaro arboviruses in the Jaci Paraná district, in the municipality of Porto Velho, capital of the state of Rondônia state, Brazil^7^. *Mansonia pseudotitillans* has been found to be naturally infected with the Saint Louis encephalitis virus^8^ *.*

In Brazil, however, there is no record of infection in people and domestic animals caused by etiological agents transmitted by *Mansonia* mosquitoes. Recently, a new virus of the family Tymoviridae in *Mansonia* spp*.* was registered in the area surrounding the Jirau Hydroelectric Dam, Rondônia, Brazil^9^. 

*Mansonia* females have aggressive hematophagous behavior during their nocturnal activity, which causes great discomfort to human populations and other animals^1^ ^,^ ^5^ ^,^ ^6^ ^,^ ^10^. In the Amazon, Tadei^10^ reported that, of all the mosquitoes collected after filling the Tucuruí Reservoir (state of Pará, Brazil), 97.1% were *Mansonia* spp., with a predominance of *Ma. titillans* (96.0%), and an average of 612 mosquitoes performing hematophagy per man/hour. The environmental impacts that occurred in the area surrounding the Jirau Hydroelectric Dam^10^ and the reduction in water flow in their breeding sites in the municipality of Porto Velho, Rondônia state, Brazil, caused the proliferation of *Mansonia* spp. due to the abundance of aquatic plants such as *Eichornia crassipes* (Mart.) Solms-Laubach, *Eichornia azurea* (Sw.) Kunth, *Pistia* sp., *Salvinia* sp., and others^1^ ^,^ ^4^.

DNA barcoding for individuals of *Mansonia* spp. that were captured in 15 locations in the area surrounding the Jirau Hydroelectric Dam showed cryptic speciation within *Mansonia dyari* and near cryptic speciation for *Mansonia titillans* ^11^; however, the taxonomic differentiation between *Mansonia* spp. remains difficult.

Classic cytogenetics is a useful tool for understanding the questions regarding chromosome structure, chromosomal evolution, and cytotaxonomy, principally for mosquitoes of medical importance^12^. Further studies for addressing questions such as the population structure, transmission capacity, and genomic evolution of mosquitoes depend on greater knowledge of chromosome structure and function. Chromosomal karyotypes have been reported for 20 genera and more than 400 species in the Culicidae family^12^. However, cytogenetic data for the *Mansonia* group are rare. Currently, *Ma. uniformes* of the subgenus *Mansonioides* of Pakistan is the only karyotyped (2n=3) species^13^. 

*Mansonia humeralis*, *Ma. titillans*, *Ma. pseudotitillans*, and *Ma. indubitans* sampled in the western Amazon region present very similar phenotypic characters^1^ ^,^ ^6^ ^,^ ^14^. These species have not been karyotyped nor have they had their constitutive heterochromatin (C-banding) registered. Thus, this is the first known study of the karyotype and distribution of C-banding patterns for better understanding of the chromosomal morphology and variability of these *Mansonia* species in Brazil.

The collection of specimens was authorized by the Instituto Chico Mendes de Conservação da Biodiversidade and the Sistema de Autorização e Informação em Biodiversidade, Brazil via the permanent license number 32941.

In February, August, and November 2019, 202 larvae were captured: *Ma. humeralis* (n= 67)*, Ma. titillans* (n= 49)*, Ma. pseudotitillans* (n= 46)*,* and *Ma. indubitans* (n= 40) at eight sites in Nova Mutum Paraná and Jaci Paraná, both districts of the municipality of Porto Velho, state of Rondônia, Brazil.

To obtain the larvae, aquatic macrophytes such as *Eichhornia crassipes* and *Pistia stratiotes* were collected on the lake around the area of the Jirau Dam. The macrophytes were washed until the larvae detached from the roots. The larvae were packed in 1-liter containers, which contained *Salvinia auriculata* and water for transport to the Malaria and Dengue Vectors Laboratory of the National Institute for Amazonian Research in Manaus, state of Amazonas. All samples were morphologically identified using taxonomic keys^1^ ^,^ ^6^ ^,^ ^14^ and a stereomicroscope (Stemi 2000-C, Carl Zeiss, Germany). 

For the preparation of the mitotic chromosomes, the spreading method was used, in which 30 slides for each species containing one brain ganglia from each larva was dissected. A total of 10 with well-distended chromosomes were used for the karyotyping of each species, which were stained with Giemsa (8% phosphate buffer, pH 6.8), and another 10 slides were used to perform the C-banding method. The slides were dipped in buffer solutions (HCL 0.2 N; barium hydroxide 5%), citrate and sodium chloride - 2xSSC (sodium citrate 0.03 M: NaCL 0.3 M, pH 6.8), and then stained with Giemsa 2% in phosphate buffer, pH 6.8. For the permanent mounting of fixed slides for microscopy, we added a drop of Entellan^TM^ (107960-Merck Millipore, Darmstadt, Germany), an anhydrous mounting medium.

The mitotic chromosomes were photographed using an Carl Zeiss AxioCam MRc camera, Oberkochen, Germany, that was coupled to an Axio Imager A2 light microscope, using 100x immersion lens and Zeiss Zen software (Blue Edition version, Germany). Chromosomal measurements were taken from the centromere to the ends of the chromosomal arms, using the program Image J (National Institutes of Health and the Laboratory for Optical and Computational Instrumentation, Wisconsin, EUA). The arm ratio was calculated (AR = longer arm/shorter arm) as well as the relative size (RS% = absolute size of each chromosome x100/total size of haploid complement chromosomes), and chromosomal pairs were numbered from I to III and classified according to the usual nomenclature for Culicidae^12^. 

Karyotype analysis from brain ganglia of the larvae (n=10) for each species (*Ma. humeralis*, *Ma. titillans*, *Ma. pseudotitillans*, and *Ma. indubitans*) showed a diploid set of the preserved metaphase chromosomes (2n=6), which is similar to most mosquito species. Metaphase chromosome lengths were calculated with the mean value of all measurements for each chromosome: chromosome I was the shortest, chromosome II was intermediary, and chromosome III had the greatest length of the three chromosomes (Figure 1A-D). With the data of the mean lengths of the chromosomes, a histogram was also constructed to show the differences in chromosome lengths in ascending order of size. Figure 1E shows a slight difference in length between the chromosomes of the diploid complement of *Ma*. *indubitans* compared to those of *Ma. humeralis*, *Ma. titillans,* and *Ma. pseudotitillans.*

**FIGURE 1:**
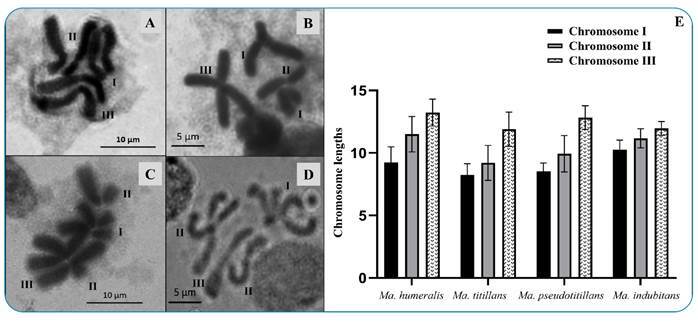
Karyotypes of mitotic metaphase chromosomes of brain ganglia of larvae of four mosquito species stained with 8% Giemsa-phosphate buffer, pH 6.8 (A-D). **(A)** *Ma. humeralis* (scale bar = 10 µm); **(B)** *Ma. titillans* (scale bar = 5 µm); **(C)** *Ma. pseudotitillans* **(**scale bar = 10 µm); **(D)** *Ma. indubitans* (scale bar = 5 µm). **(E)** Histogram of the mean lengths of the chromosome pairs I, II, and III.

The relative lengths and calculated centromeric indices of *Ma. humeralis*, *Ma. titillans,* and *Ma. pseudotitillans* chromosomes showed that pair I, sexual (XX), and II autosomal are submetacentric chromosomes, and that pair III is metacentric (Table 1, Figure 1A, B and C ). However, *Ma. indubitans* presented three metacentric pairs (Table 1, Figure 1D). 

**TABLE 1:** Mean lengths and quantitative characterization of each chromosome of four *Mansonia* mosquito species (*Ma. indubitans*, *Ma. humeralis*, *Ma. titillans,* and *Ma. pseudotitillans*).

Species	Chromosome	Length	Arm ratio	Relative size (%)	Classification
*Ma. indubitans*	I	9.90 ± 0.10	1.40 ± 0.10	30.5 ± 0.10	Metacentric
	II	10,5 ± 1.50	1.50 ± 0.20	32.4 ± 0.30	Metacentric
	III	11.3 ± 1.00	1.60 ± 0.10	35.1 ± 0.70	Metacentric
*Ma. humeralis*	I	9.14 ± 1.20	1.80 ± 0.60	27.0 ± 1.40	Submetacentric
	II	11.2 ± 1.40	2.00 ± 0.20	33.1 ± 1.60	Submetacentric
	III	13.5 ± 2.60	1.30 ± 0.20	39.8 ± 2.70	Metacentric
*Ma. titillans*	I	8.30 ± 1.60	1.60 ± 0.60	28.7 ± 1.00	Submetacentric
	II	9.20 ± 0.40	1.60 ± 0.40	31.8 ± 1.40	Submetacentric
	III	11.4 ± 2.00	1.10 ± 0.10	39.4 ± 1.60	Metacentric
*Ma. pseudotitillans*	I	8.60 ± 0.80	1.80 ± 0.30	27.3 ± 0.70	Submetacentric
	II	9.90 ± 1.30	2.10 ± 0.40	31.8 ± 1.40	Submetacentric
	III	12.8 ± 1.00	1.10 ± 0.30	40.5 ± 0.70	Metacentric

In *Ma. humeralis,* a difference of 2 µm was observed in the mean length of chromosome I (9.14 µm), chromosome II (11.2 µm), and chromosome III (13.5 µm). For *Ma. indubitans,* these values differed by 1 µm, with the chromosomes measuring 9.90, 10.5, and 11.3 µm, respectively. *Mansonia humeralis* also presented a greater mean haploid genome length (33.1 ± 1.90 µm) when compared to *Ma. titillans* (28.9 ± 0.91 µm) (Table 1).

The C-banding method in chromosomes of the brain ganglia (n=10) for *Ma. humeralis, Ma. titillans, Ma. psdeudotitillans,* and *Ma. indubitans* showed standard centromeric heterochromatin blocks. In addition, compaction and distribution levels of interstitial and telomeric C-bands, with intra-individual and interspecific variation in autosomal and sex chromosomes, were recorded (Figure 2). *Mansonia humeralis* presented centromeric and pericentromeric C-bands in the short arms of the autosomal and sexual pairs (Figure 2A, B ). *Mansonia titillans* showed weak or no centromeric heterochromatin in autosomal and sexual pairs (Figure 2C, D ). In *Ma. pseudotitillans,* variations in the condensation and localization of the constitutive heterochromatin occurred in the centromeric regions of chromosomes I, II, and III, interstitial regions of the short arm of the sex chromosome, and subtelomeric regions of the long arm of the autosome III (Figure 2E, F ). *Mansonia indubitans* showed strongly stained C-band segments in the centromere of pairs I, II, and III, and it also showed variation in the intensity of the staining in the telomere of the long arm of pair II and telomeres of the sexual pair (Figure 2G, H ). 

**FIGURE 2:**
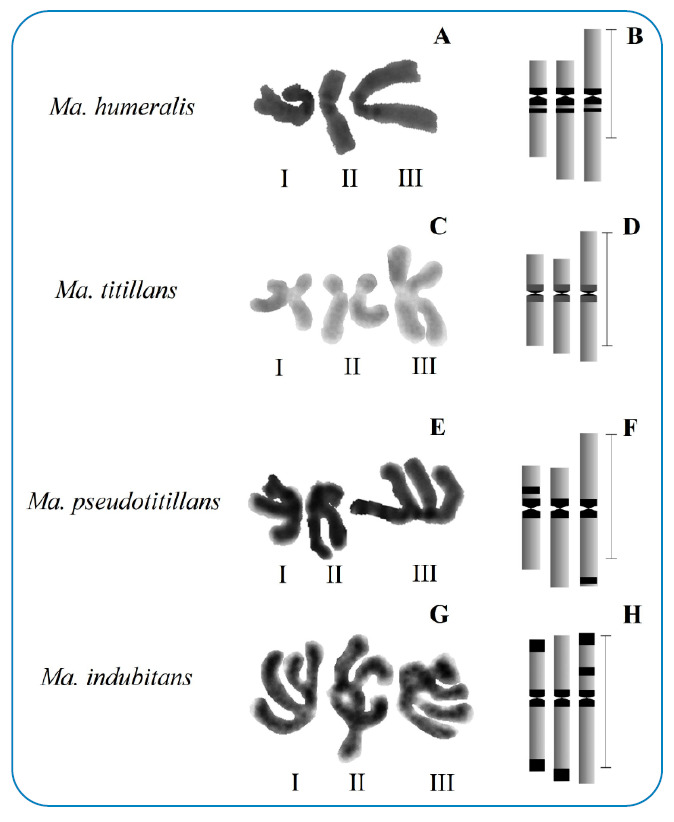
**(A)** *Ma. humeralis*; C - *Ma. titillans*; E - *Ma. pseudotitillans*; G - *Ma. indubitans.* Metaphase nuclei of brain ganglia of larvae subjected to the **(C)** banding method, and histogram of constitutive heterochromatin blocks and their distribution in the chromosomes (I, II and III) **(B, D, F, and H)**, respectively. Arrows indicate constitutive heterochromatin blocks in the centromeric, pericentromeric, and subtelomeric regions of the arms of autosomal and sex chromosomes. **Scale: B, D, F, H** = 10 µm.

Herein, we described for the first time the morphology, lengths, and proportions for the metaphase chromosomes of *Ma. humeralis, Ma. titillans, Ma. pseudotitillans*, and *Ma. indubitans*. The measurements showed that the diploid number of these species remained at six in the family Culicidae^12^.

*Mansonia indubitans* presented metacentric chromosomes in the three pairs of chromosomes and it was the species with the centromeric profile that was most similar to the metaphase chromosomes of *Ma. uniformes* ^13^, which is the only karyotyped species of the subgenus *Mansonioides*. *Mansonia indubitans* has pairs I and III that are metacentric and pair II that is slightly submetacentric. However, *Ma. humeralis*, *Ma. titillans,* and *Ma. pseudotitillans* have submetacentric pair I and II chromosomes and a metacentric pair III. The sex chromosomes were homomorphic (XX), i.e., metacentric for the four species analyzed in this study.

The levels of compaction and localization of C-bands occurred in the centromeric, interstitial, and telomeric regions of the autosomal and sexual chromosomes of these *Mansonia* species. The chromosomal variability of mosquitoes can be caused by differences in inversions that occur in the homologous chromosomes of the same individual, as in *Anopheles darlingi* ^15^. Intraspecific variation of heterochromatin blocks recorded in mitotic chromosomes of *Ma. humeralis, Ma. titillans, Ma. pseudotitillans,* and *Ma. indubitans* does not necessarily mean that possible inversions alone distributed such C-band blocks in the chromosomes. The accumulation and distribution of these blocks of constitutive heterochromatin in these mosquitoes, originating in the affected area of the hydroelectric dam, may have occurred due to cumulative differences of repetitive DNA cistrons throughout the evolutionary process and / or even environmental changes. 

This study is a milestone in the karyotypic description, location, and distribution of constitutive heterochromatin blocks at different levels of compaction in the chromosomes of *Ma. humeralis, Ma. titillans, Ma. pseudotitillans,* and *Ma. indubitans* from the area of the Jirau Hydroelectric Dam, state of Rondônia, Brazil. These data are also useful for cytogenetic characterization of these mosquitoes at the species level, and are extremely relevant for the understanding of intraspecific chromosomal variability and for cytotaxonomic characterization, and can be used in studies aimed at control programs for these mosquitoes, which are able to transmit arboviruses and lymphatic filariasis to humans and other vertebrates.
